# 24-Hour Ex Vivo Hypothermic Acellular Perfusion of Porcine Forelimb: A 7-Day Follow-up Study

**DOI:** 10.1097/PRS.0000000000011469

**Published:** 2024-04-15

**Authors:** Kaj Brouwers, Anne Sophie Kruit, Dominique van Midden, Her J. H. Zegers, Jonne Doorduin, Erik Koers, Stefan Hummelink, Dietmar J. O. Ulrich

**Affiliations:** Nijmegen, the Netherlands; From the Departments of 1 Plastic and Reconstructive Surgery; 2 Pathology; 3 Cardiothoracic Surgery; 4 Neurology, Donders Institute for Brain, Cognition, and Behaviour, Radboud University Medical Center.

## Abstract

**Background:**

One of the limiting factors for vascularized composite allograft storage is the short viable ischemic time (4 to 6 hours). Hypothermic machine perfusion enables near-physiologic preservation, avoiding the deleterious effects of hypoxia and static cooling. This study aims to compare muscle injury after 24-hour acellular perfusion with static cold storage (SCS) in a porcine limb replantation model, examining outcomes for up to 7 days after reperfusion.

**Methods:**

Sixteen procured porcine forelimbs were perfused under hypothermic conditions for 24 hours with histidine-tryptophan-ketoglutarate (*n* = 8) or preserved on ice for 4 hours (SCS; *n* = 8) before heterotopic replantation. Muscle injury was assessed using biochemical markers, and muscle biopsies were analyzed using the Histologic Injury Severity Score.

**Results:**

During preservation, limb weight decreased by 2% in the SCS group and increased by 44% in the perfusion group (*P* < 0.001). Twelve limbs (histidine-tryptophan-ketoglutarate, *n* = 6; SCS, *n* = 6) survived for 7 days. Three days after replantation, increased creatinine kinase levels were observed in the perfusion group (33,781 versus 2163 mmol/L; *P* < 0.001). The mean end point Histologic Injury Severity Score was 3.8 (SD 0.7) in the perfusion group and 1.8 (SD 0.7) in the SCS group (*P* = 0.008), mostly due to increased edema (*P* = 0.004).

**Conclusion:**

A total of 24 hours of hypothermic machine perfusion and 4 hours of SCS of the vascularized composite allograft demonstrated minimal degenerated muscle tissue 7 days after replantation.

**Clinical Relevance Statement:**

This project will widely advance the field of reconstructive research and provide strong preclinical data to allow human clinical trials with great potential to change the standard of care in reconstructive transplantation.

Vascularized composite allografts (VCAs) have become an essential therapeutic solution for many patients with facial defects, upper extremity amputations, or major composite tissue defects not adequately addressed by autologous surgery.^1^ Technical success has been achieved in hand,^2^ face,^3^ lower extremity,^4^ and penile transplantation,^5^ with more than 150 patients having undergone vascularized composite allotransplantation procedures worldwide.^6^ However, it is challenging to match the donor and recipient. Donor criteria, transplant candidate selection, and geographic span for VCA procurement are center-specific and coordinated at a local level.^7^ Seventy percent of the 28 VCAs performed between 2014 and 2017 in the United States at 14 transplant centers were procured approximately 100 miles away from the recipient hospitals.^7^ Whereas indications for the use of VCA grow wider with the establishment of new VCA transplant centers,^8^ the limited viable ischemic time of VCA remains a major obstacle to its broader application.^9^

The standard in VCA preservation is based on static cold storage (SCS): cooling the tissue with a cold preservation solution and storing it in a box on a 4°C ice slurry for up to 4 to 6 hours before transplantation.^10^ The principle of SCS is to minimize the deleterious effects of ischemia and lack of nutrients during the preservation interval. Under these hypothermic conditions, metabolic reactions are considerably slower.^11^ Disadvantages include the time-dependent injury caused by hypothermia, the lack of real-time assessment of graft viability, and restricted ex vivo preservation times, limiting the radius for VCA procurement.^7,12^

In the past 2 decades, machine perfusion (MP) has proven to be a reliable preservation method for solid organs and has become clinical routine.^13,14^ This technique allows for continuous and controlled oxygenation and aims at partly maintaining subphysiologic cellular metabolism while avoiding cold injury due to hypoxia, reducing ischemia-reperfusion injury (IRI) and the risk of graft rejection.^15,16^ MP has shown promising results for VCA preservation, and effectively increased tissue preservation to 12 or 24 hours in rodents,^17,18^ swine,^19–24^ and human limb models.^25,26^ These studies promote a new approach to the management of donor VCAs by expanding the current boundaries of VCA. However, these studies do not include reperfusion outcomes or are limited by short-term survival.

To address these challenges, our focus was on investigating the effect of ischemia-reperfusion on skeletal muscle up to 1 week after reperfusion, alongside examining various hemodynamic and perfusion measures. In this noninferiority porcine whole-limb replantation study, it was hypothesized that 24 hours of hypothermic MP would show comparable biochemical and histologic muscle alterations compared with 4 hours of SCS after 7 days follow-up.

## PATIENTS AND METHODS

### Study Design

A heterotopic forelimb replantation model in pigs was established. Before surgery, animals were assigned randomly to 1 of 2 experimental groups. In the SCS group (control, *n* = 8), forelimbs were procured and stored in dry gauze in a sealed bag on ice slurry at 4°C for 4 hours and underwent subsequent heterotopic replantation. In the MP group (treatment, *n* = 8), surgical amputation of the forelimb was followed by ex situ hypothermic MP for 24 hours and subsequent heterotopic replantation. After the surgical procedure, the animals were housed under standard conditions with access to food and water ad libitum and monitored for 7 days before the animals were euthanized with an overdose of phenobarbital. All surgical procedures and measurements were performed by the same researcher (K.B.).

### Animal Model

Sixteen female Dutch Landrace pigs (weight 40 to 50 kg) were used for this study. All animal experiments were conducted in compliance with the local animal experimentation committee of the Radboud University Medical Center and the national animal experimentation committee (2021-0039-001) and were in accordance with Animal Research: Reporting of In Vivo Experiments guidelines.

### Limb Procurement

The pig was positioned in right side recumbency. The left forelimb was incised through a semicircular axillary incision, followed by a dissection of the axillary neurovascular bundle similar to previously described techniques. (**See Figure, Supplemental Digital Content 1**, which provides color images of the surgical procedure. [*Above*, *left*] The pig is positioned in right side recumbency. [*Above*, *right*, and *center*, *left*] The left forelimb was incised through a semicircular axillary incision, followed by a dissection of the axillary neurovascular bundle. [*Center*, *right*] The shoulder muscles were cut circumferentially, a fasciectomy of the flexor and extensor muscles distally of the elbow was performed to prevent compartment syndrome, and the limb was harvested. [*Below*, *left*] After amputation, the artery was cannulated with a 10- to 12-Fr sheath and flushed with 1 L of heparin–histidine-tryptophan-ketoglutarate [HTK] solution [200 E heparin per 100 mL of HTK solution]. [*Below*, *right*] Eight forelimbs were attached to a custom-made open extracorporeal circuit and perfused for 24 hours with 2 L of HTK, http://links.lww.com/PRS/H192.)^20,22–24^ The shoulder muscles were cut circumferentially, a fasciectomy of the flexor and extensor muscles distally of the elbow was performed to prevent compartment syndrome, and the limb was procured. After amputation, the artery was cannulated with a 10- to 12-Fr sheath and flushed with 1 L of heparin–HTK solution (200 E heparin per 100 mL of modified HTK solution) for 15 minutes.

### Extracorporeal Setup and Perfusion Protocol

**Fig. 1.**
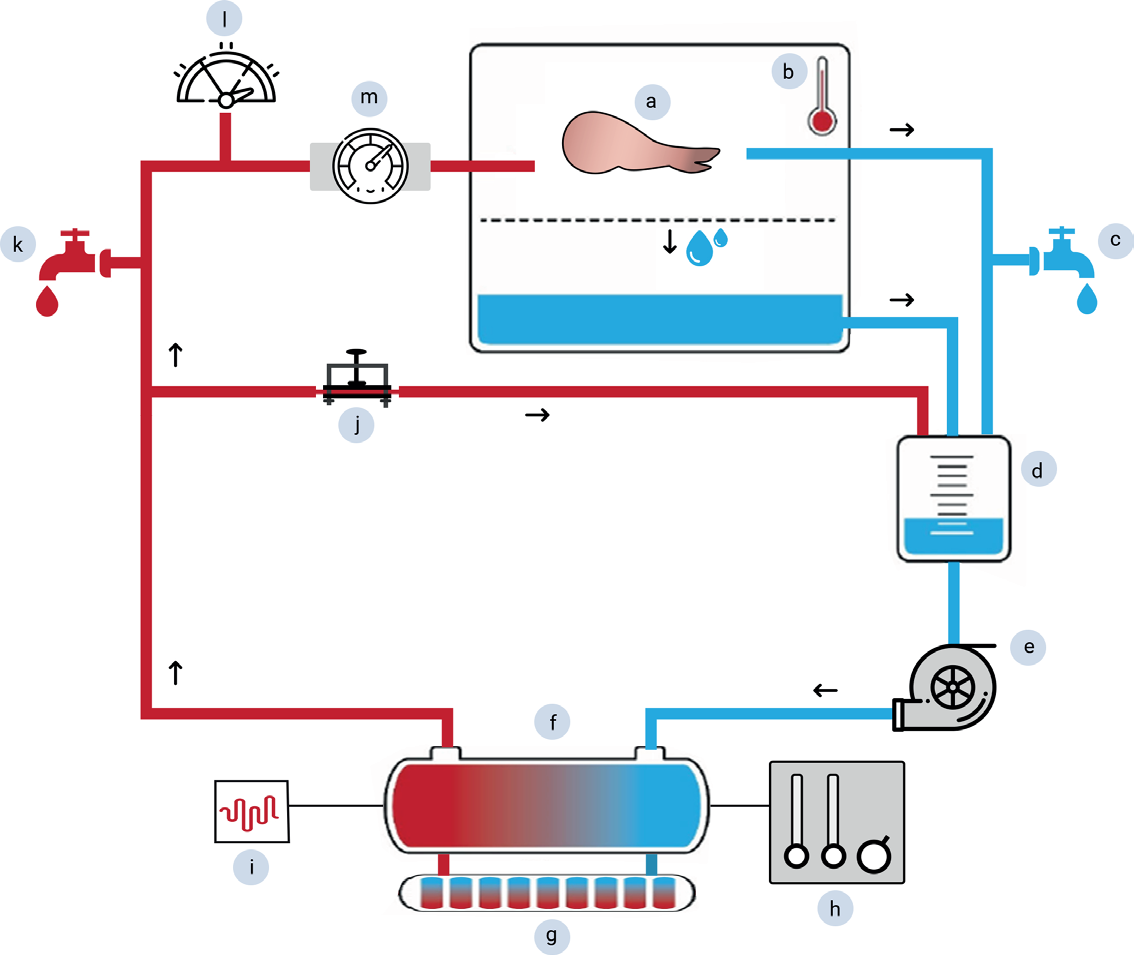
Schematic illustration of the custom-made extracorporeal perfusion setup. The semiclosed perfusion setup includes (*a*) the porcine limb in the perfusion box on top of a metal grid, (*b*) a 15-mm needle probe measuring muscle temperature, passive venous drainage of the preservation fluid and (*c*) venous sampling point, (*d*) collection reservoir, (*e*) centrifugal pump, (*f*) heater–cooler machine cooling the solution to 8°C to 10°C, (*g*) oxygenator, (*h*) infusing the fluid with a mix of 95% O_2_ and 5% CO_2_, and (*i*) a monitor for pressure and saturation readings. A bifurcated tubing line (*j*) was connected to provide high flow directly back into the venous reservoir and a controllable flow to the canulated limb’s pedicle. (*k*) Drug administration point/fluid sampling point, (*l*) ultrasonic flow meter, (*m*) arterial pressure measuring an in-line pressure between 25 and 30 mm Hg are shown. Perfusate samples are collected through a pregraft sampling point and a cannulated vein after grafting.

Forelimbs were attached to a custom-made open extracorporeal circuit to provide oxygenated, continuous, and nonpulsatile flow according to previous studies performed by the same research group^27–29^ (Fig. 1). All limbs were perfused for 24 hours with 2 L of HTK (Custodiol; Koehler Chemi) enriched with 40 mg of methylprednisolone, glucose 50% (1 mL/L), insulin (10 IE/L), polyethylene glycol (20 g/L), and L-glutamine (10 mL/L).^30–32^ The perfusion solution was gradually cooled down to 8°C according to the manufacturer’s recommendations. Flow was measured continuously using an ultrasonic flow meter (SonoTT ultrasonic flow computer; EmTec). The centrifugal pump (BP-80 Bio-Pump Centrifugal Blood Pump; Medtronic) was regulated to maintain an in-line pressure between 25 and 30 mm Hg. The perfusion solution was not replenished throughout the entire perfusion period. MP measures (flow, pressure, pump speed, and temperature) were assessed hourly during perfusion.

### Measurements

Limb viability assessments (capillary refill times, temperature, skin color, and necrosis) were carried out at baseline and 1, 3, and 7 days after replantation. Limbs were weighed after harvest, after the ex vivo preservation period, and at 7 days after replantation, as both IRI and hypothermic perfusion could potentially contribute to weight increase of the vascularized composite tissue.^10^ Limb perfusion was appraised using a near-infrared fluorescence angiography camera (PhotoDynamic Eye; Hamamatsu Photonics) that recorded the surgical field after each intravenous injection of 5 mg/mL indocyanine green fluorescent marker (Verdye; Diagnostic Green GmbH). Near-infrared fluorescence angiography was performed after limb harvest, directly after replantation, and 3 and 7 days after replantation. To assess muscle function, nerve conduction studies of the median nerve with the active electrode in the flexor carpi radial muscle were performed at baseline, after replantation, and at postoperative days 3 and 7.

### Specimen Procurement

Muscle biopsy specimens, blood draws from the systemic circulation, and perfusate aliquots before and after passing through the tissue were obtained to assess ischemia-related muscle damage. Blood and perfusate analysis was performed using CG4+ and CG8+ cartridges to measure pH, lactate, oxygen partial pressure, and carbon dioxide partial pressure using the iSTAT platform (Abbott). Potassium and creatine kinase (CK) were measured in a heparinized syringe using a 1265 Rapidlab Blood Gas Analyzer (Siemens Healthcare Diagnostics). Vascular resistance was measured as pressure divided by flow. Oxygen consumption was calculated according to the Fick formula:


$$\begin{array}{l} Oxygenconsumption=\left(\Delta pO_{2}\times So_{2}\times L_{perf}\right)/O_{wt} \end{array}$$

where ΔpO_2_ is the difference between the pO_2_ of the delivered HTK and the pO_2_ of the limb outflow (in mm Hg), So_2_ is 370 nM O_2_/mL (oxygen solubility per milliliter of solution at 5°C at a barometric pressure of 760 mm Hg),^33^ L_perf_ is limb perfusion (in milliliters per minute), and O_wt_ is limb weight (in grams).

Muscle biopsy specimens were acquired from a flexor and extensor muscle of the limbs at the beginning of the amputation surgery, every 6 hours during ex situ preservation, and 1, 3, and 7 days after replantation (under sedation). Perfusate aliquots were obtained every 2 hours during perfusion and blood samples at the beginning of the amputation surgery, 0 hours, and 1, 3, and 7 days after replantation. (**See Table, Supplemental Digital Content 2**, which provides an overview of all measurements during the experiments, http://links.lww.com/PRS/H193.)

### Histologic Analysis

Formalin-fixed biopsy specimens were sectioned and stained with hematoxylin and eosin (H&E) and digitized using a Panoramic P1000 whole slide scanner (3DHISTECH) at a resolution of 0.24 µm per pixel. Ten randomly selected high-power fields from each cross-sectional biopsy specimen were assessed for damage to muscle fibers by a pathologist (D.v.M.) blinded to the intervention groups. Histologic Injury Severity Score (HISS; range, 0 to 12; Table 1) was used to assess for IRI-induced alterations, according to methods previously described by our research group.^19,34–36^

**Table 1.** Histology Injury Scoring System for Hypoxia-Induced Muscle Injury

Morphologic Changes	Categories
Interstitial edema	0: No significant increase 1: Minimal 2: Intermediate 3: Severe/diffuse
Inflammation	0: Not significant 1: Minimal 2: Intermediate 3: Diffuse
Variation in shape and size of myocytes	0: Homogeneous 1: Mild heterogeneous 2: Intermediate heterogeneous 3: Severe heterogeneous
Damaged muscle fibers^a^	0: 0–5 myocytes/10 HPF (20× magnification) 1: 6–20 myocytes/10 HPF (20× magnification) 2: 21–50 myocytes/10 HPF (20× magnification) 3: >51 myocytes/10 HPF (20× magnification)
Total score	0–4: No/minimal degeneration 5–7: Intermediate degeneration 8–12: Severe degeneration

HPF, high-power field.

a Damaged muscle fibers: a sum of necrotic, hypoxic, and phagocytic myocytes.

### Statistical Analysis

To detect significant differences in HISS between MP and SCS groups, a sample size of 8 limbs per study arm was estimated based on previous MP experiments using porcine limbs,^19,34^ with a standardized effect size of 1.2 at a 2-sided α of 0.05 and 80% power. Continuous data are presented as mean (SD). Data analysis was performed using SPSS 29.0 (IBM Corporation) and Microsoft Excel 2016 (Microsoft Corp.). For comparison between 2 groups at different time points, either a Mann-Whitney *U* test or mixed-effect analysis was performed. In the case of multiple comparisons, *P* values were adjusted by Tukey correction. Statistical significance was defined by a *P* value less than 0.05.

## RESULTS

Baseline characteristics were comparable between the intervention groups (Table 2). Warm ischemia time before storage was 10.3 minutes longer in the MP group compared with the SCS group (*P* = 0.005), as a result of cannulating, flushing, and attaching the limb to the perfusion machine.

**Table 2.** Baseline Characteristics of the Static Cold Storage Group and the Machine Perfusion Group

Characteristics	SCS (*n* = 8), Mean (SD)	MP (*n* = 8), Mean (SD)	*P*
Harvest, min	92 (17)	87 (14)	0.636
Flush, min	15 (0)	15 (0)	1.000
Ex vivo storage time, hrs	4 (0)	24 (0)	<0.001
WIT before storage, min	2.6 (1.1)	12.9 (7.4)	0.005
WIT before reperfusion, min	52 (27)	58 (16)	0.172
Limb weight before intervention, g	2580 (285)	2554 (395)	0.916
Limb weight after intervention, g	2542 (274)	3676 (312)	<0.001
Weight difference, %	−2	44	<0.001
Temperature before intervention, °C	35.4 (1.0)	34.6 (1.1)	0.171
Temperature after intervention, °C	6.5 (1.5)	12.0 (1.4)	<0.001

WIT, warm ischemia time.

### MP Results

**Fig. 2.**
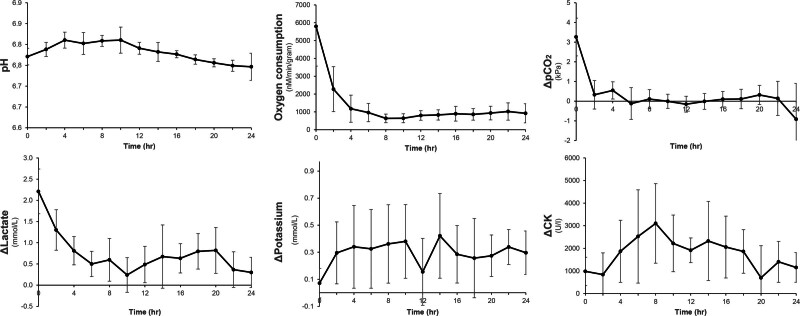
Mean perfusate biochemical measures during 24 hours of ex vivo perfusion of porcine limbs with 95% CIs.

Mean limb core temperature dropped to 12.0°C (SD 1.4) within the first 6 hours of perfusion and to 6.5°C (SD 1.5) in 4 hours of SCS (*P* < 0.001). During MP, the average flow was 23.7 mL/min (SD 12.6). Vascular resistance increased over time from 0.45 mm Hg × min/mL at baseline to 2.44 mm Hg × min/mL at the end of the perfusion (*P* < 0.001). (**See Figure, Supplemental Digital Content 3**, which shows the mean perfusion parameters measured hourly during continuous oxygenated MP with modified HTK solution [*n* = 8]; flow [mL/min], pressure [mm Hg], vascular resistance [mm Hg × min/mL], and temperature [°C], with 95% CIs, http://links.lww.com/PRS/H194.) Mean immediate oxygen consumption was noted (at T0 hours, 5799; SD 322 nM/min/g) and decreased over time, reaching a stable level (T24 hours, 921; SD 212 nM/min/g) (Fig. 2). Overall weight gain at the end of perfusion was statistically significant compared with limb weight before MP (*P* < 0.001). Macroscopically, edema tended to develop mostly in the subcutaneous layer.

The release of potassium in the perfusate increased from an initial 0.07 mmol/L (SD 0.16) to 0.3 mmol/L (SD 0.23) at the end of perfusion (*P* = 0.227). Release of CK increased from 982 mmol/L (SD 899) at baseline and peaked at 3102 mmol/L (SD 2539) at 8 hours of perfusion (*P* = 0.068), after which it showed a decline until the end of the perfusion. Lactate production peaked at the beginning of perfusion but remained less than 2 mmol/L on average at the end of 24 hours of perfusion. This did not change the overall perfusate pH (mean 6.78, SD 0.02).

### After Reperfusion

#### Clinical Data

Out of 16 experiments, 4 animals did not provide data for postoperative day 7 due to vascular pedicle avulsion leading to death (SCS group: *n* = 1, postoperative day 4), thrombotic event (SCS group: *n* = 1, venous thrombosis, postoperative day 5; MP group: *n* = 1, arterial thrombosis, postoperative day 4), or reaching humane end point (MP group: *n* = 1, hind limb arterial thrombosis after removing inguinal atrial cannula, postoperative day 3). Animals undergoing heterotopic replantation recovered well from general anesthesia and maintained hemodynamic and respiratory stability throughout the entire 7-day observation period. Animals in the SCS group were able to stand up independently on 3 legs 17.8 hours (SD 6) after replantation, compared with an average of 32.3 hours (SD 18.9) in the MP group (*P* < 0.001). After 1 week, limb viability assessments (capillary refill times, temperature, skin color) were comparable with baseline measurements. (**See Figure, Supplemental Digital Content 4**, which provides color images of perfused and static cold-stored limbs at baseline, directly after replantation, and 3 and 7 days after replantation. After a week, limb viability assessments (capillary refill times, temperature, skin color) were comparable with baseline measurements. Below is an appraisal of limb perfusion using near-infrared fluorescence angiography, showing intact circulation, http://links.lww.com/PRS/H195.) Three limbs (SCS, *n* = 1; MP group, *n* = 2) showed (partial) skin necrosis (1% to 5%) at 7 days after replantation. Indocyanine green angiography confirmed microcirculation of all autografts, with no signs of venous or arterial occlusions throughout the 7-day follow-up period. (**See Video [online]**, which provides an assessment of perfusion using near-infrared fluorescence angiography in an SCS and perfusion limb on postoperative days 3 and 7.)

**Video.** This video provides an assessment of perfusion using near-infrared fluorescence angiography in an SCS and perfusion limb on postoperative days 3 and 7.

#### Blood Gases

**Fig. 3.**
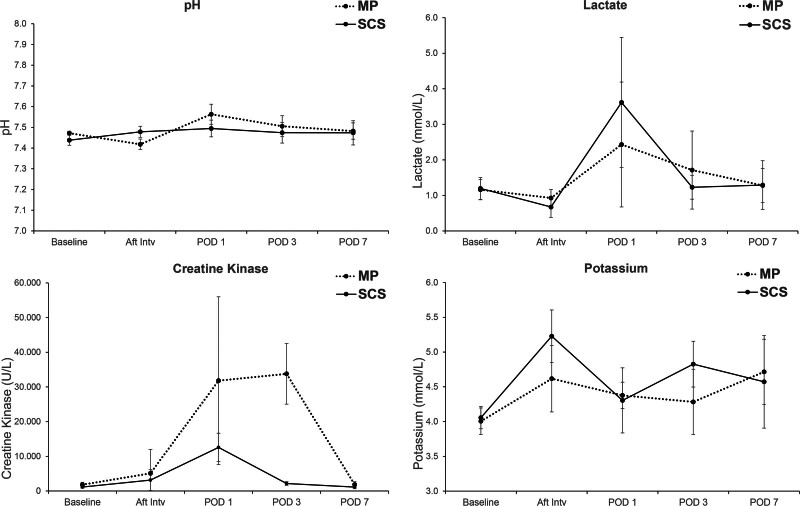
Biochemical measures at baseline (MP, *n* = 8; SCS, *n* = 8), after intervention (MP, *n* = 8; SCS, *n* = 8), postoperative day (POD) 1 (MP, *n* = 8; SCS, *n* = 8), postoperative day 3 (MP, *n* = 8; SCS, *n* = 8), and postoperative day 7 (MP, *n* = 6; SCS, *n* = 6); mean per group with 95% CIs.

In the first 3 days after replantation, statistically significant increased levels of muscle damage markers were observed in the MP group versus SCS; specifically, CK (33,781 versus 2163 mmol/L; *P* < 0.001) (Fig. 3). Lactate increased in both groups on the first day after replantation (2.43 versus 3.62 mmol/L; *P* = 0.372), whereas pH remained at approximately 7.46 in the perfusion group and peaked at 7.56 at 24 hours after replantation in the SCS group.

#### Electromyography

In all limbs, nerve conduction studies showed reproducible and high-amplitude compound muscle action potentials (CMAPs) in the flexor carpi radial muscle at baseline. However, a CMAP response could only be elicited in 5 limbs directly after replantation (SCS, *n* = 4; MP, *n* = 1). Three and 7 days after replantation, none of the limbs showed any CMAP response upon median nerve stimulation, and only 4 limbs showed little response after direct muscle stimulation (SCS, *n* = 3; MP, *n* = 1) 7 days postoperatively.

#### Histopathology

**Fig. 4.**
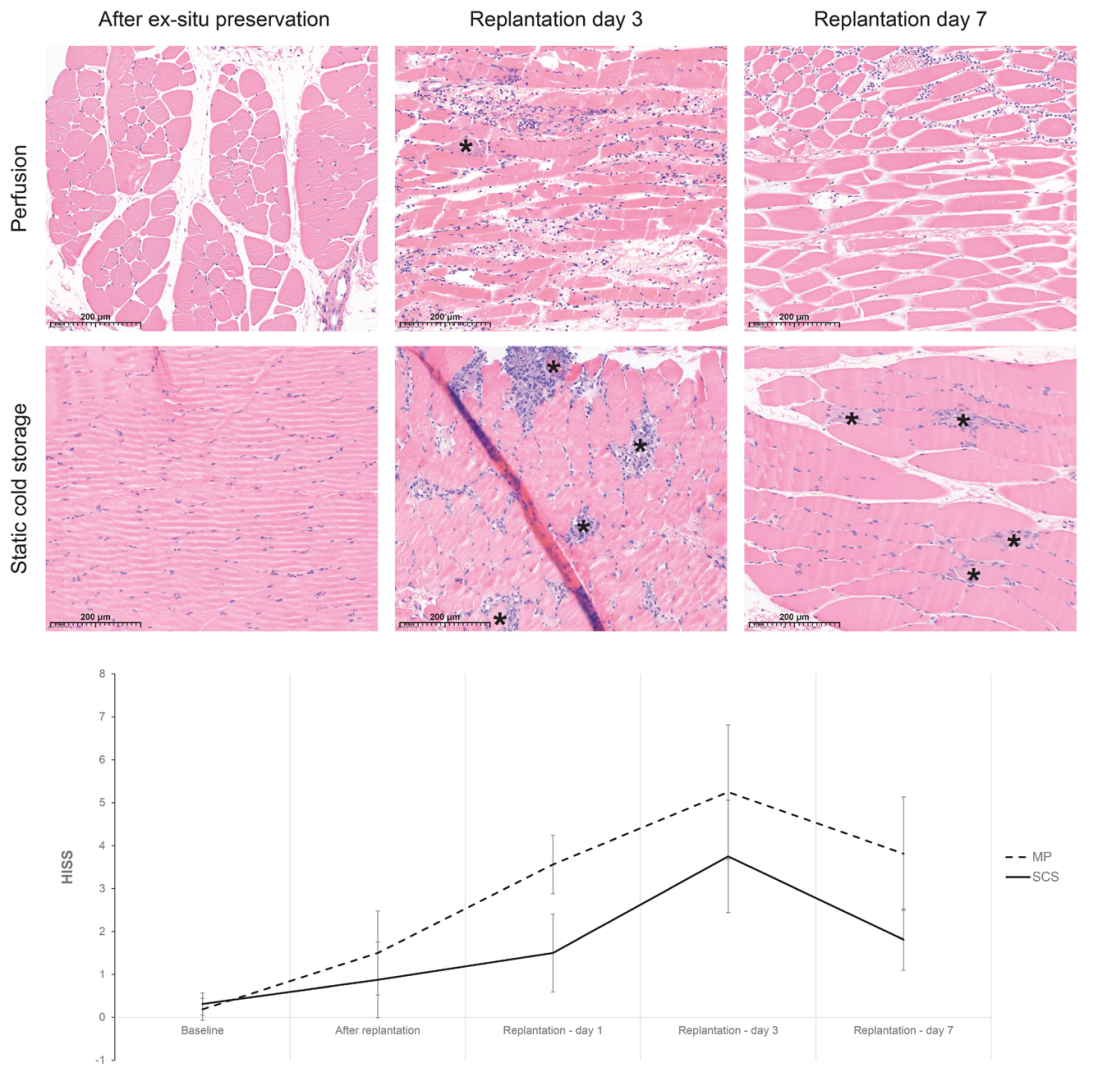
Representative H&E-stained cross-sections (magnification ×20) from perfused and static cold stored porcine flexor muscle directly after ex situ preservation, 3 and 7 days after replantation (*above*). Perfused limbs showed significantly more edema (*P* < 0.001), whereas other subscores were not significantly different between the groups 3 days after reperfusion. Seven days after reperfusion, HTK-perfused muscle showed moderate alterations. There is mild variation in muscle fiber size, mild interstitial edema, and mild to moderate interstitial inflammation without damaged muscle fibers (HISS 4). In comparison, static cold-stored muscle fibers showed mild variation in muscle fiber size, mild interstitial inflammation, and a few damaged muscle fibers (*asterisks*). No significant interstitial edema is seen (HISS 3). Total HISS in H&E-stained cross-sections of skeletal muscle biopsies. Values are shown for baseline, after replantation, and 1, 3, and 7 days after reperfusion (*below*).

**Fig. 5.**
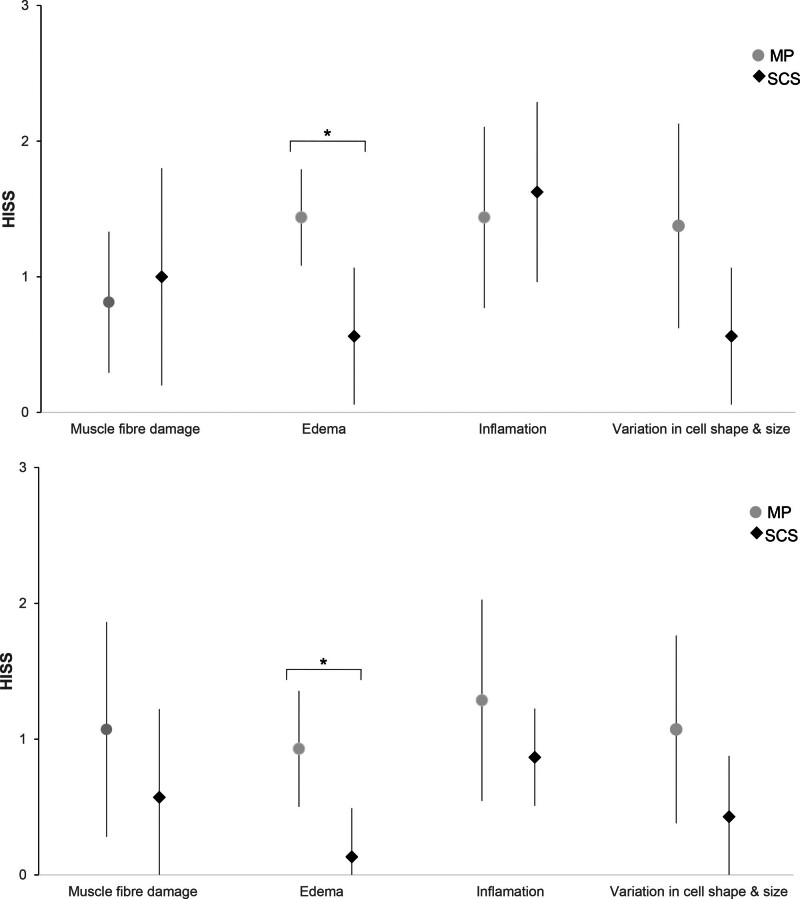
Mean sub-HISS of perfused and static cold-stored limbs at 3 and 7 days after replantation. (*Above*) 3 days after replantation (MP, *n* = 8; SCS, *n* = 8): muscle fiber damage (*P* = 0.578), edema (*P* < 0.001), variation in cell shape and size (*P* = 0.008), and inflammation (*P* = 0.615). (*Below*) Subscores at 7 days after replantation (MP, *n* = 6; SCS, *n* = 6): muscle fiber damage (*P* = 0.146), edema (*P* = 0.004), variation in cell shape and size (*P* = 0.077), and inflammation (*P* = 0.126). *Asterisks* indicate significance.

The mean HISS was 5.3 (SD 0.7) in the MP group and 3.8 (SD 0.7) in the SCS group (*P* = 0.110) 3 days after reperfusion, corresponding to intermediate and minimal degeneration, respectively (Fig. 4). MP limbs showed significantly more edema (*P* < 0.001), whereas other subscores were not significantly different between the groups (Fig. 5, *above*). Seven days after reperfusion, the mean HISS was statistically significantly different, measuring 3.8 (SD 0.7) in the MP group and 1.8 (SD 0.7) in the SCS group (*P* = 0.008), corresponding to minimal degenerated muscle tissue in both groups. This difference in histology scores resulted from a significantly higher subscore for edema (*P* = 0.004) and variation in cell shape and size (*P* = 0.077) in MP limbs. Subscores including muscle fiber damage (*P* = 0.146) and inflammation (*P* = 0.126) showed high individual variation and did not show statistically significant differences (Fig. 5, *below*).

## DISCUSSION

In this porcine whole-limb replantation model, muscle histology scores demonstrated minimal degenerated muscle tissue in both groups 7 days after replantation while extending the allowable ischemia time from 4 to 24 hours using oxygenated continuous MP with HTK. Although machine-perfused limbs showed statistically significant increased edema and variation in muscle cells compared with the SCS group 7 days after replantation, the clinical significance of these results on tissue viability is uncertain. These results align with previous studies that evaluated muscle damage up to 7 days after 24-hour MP and subsequent replantation of amputated porcine limbs. The use of MP with acellular^20,22^ and autologous blood^24^ solutions exhibited reduced histopathologic muscle tissue damage compared with static cold storage.

There is a lack of objective criteria to define the transplant quality of VCAs. In extended MP, biochemical markers such as potassium, CK, and lactate acid levels serve as recognized “real-time” measures to assess tissue damage.^16^ Consistent with our findings, several researchers have reported notable reductions in potassium and myoglobin levels after 24-hour MP during porcine limb replantation, compared with 4-hour static cold storage, despite an initial increase during MP.^19,20^ Biopsy specimens from the perfusion group displayed better-preserved muscle tissue architecture. It is hypothesized that these markers are released continuously into the perfusate/blood from the severed end of the limb, hindering the detection of smaller quantities released within the limb itself. The predictive value of biochemical markers in estimating muscle damage or functional outcomes remains to be fully understood.

Weight gain during hypothermic perfusion could be a key indicator for the assessment of limb condition. Meyers et al.^37^ demonstrated that weight gain correlated to microscopic muscle injury and was the earliest evidence of limb dysfunction during MP. In a porcine forelimb model preserved for 20 hours with a hemoglobin-based oxygen carrier (HBOC-201) using MP, 20% weight gain of the limb led to significantly lower contractility than baseline (*P* = 0.003). Apart from higher vascular resistance and lower perfusate flow in a constant pressure configuration, edema could potentially make the replantation procedure more difficult.^27,38,39^ In the presented study, a significant increase of weight of 44% was seen postperfusion, similar to other research with acellular and crystalloid solutions, varying between 10%^22^ and 41%^20^ in swine limb models. Although the scope of the effects of edema formation during perfusion on the viability of the ex situ perfused limb is a matter of question, it points to a series of responses usually formed under pathologic conditions, such as IRI.

In the clinical setting, lack of muscle contraction may be an early indicator of critical limb physiology.^26^ Despite ongoing efforts, functional testing of VCA is a major hurdle to overcome. Methods of functional evaluations, such as electromyography, were cumbersome in our experience. Muscle contractility is influenced by multiple variables, such as perfusate potassium, calcium, and pH,^26^ but also limb temperature and edema.^19^ It seems that the contractility is inversely correlated with perfusate potassium levels in the VCA directly after replantation, whereas Wallerian degeneration^40^ might negatively affect the measurement at 3 and 7 days postoperatively. To the contrary, Ozer et al.^21,41^ investigated blood-based normothermic perfusion to sustain porcine forelimbs ex vivo for 12 hours with intact neuromuscular responses up to 24 hours after replantation using a single fiber muscle contraction test. Although encouraging, this short follow-up should be interpreted within its limits, due to the longstanding process of denervation over weeks, which might worsen in the presence of IRI.

The results of the current study need to be examined in light of its limitations. First, even though equal preservation times between MP and SCS would have been ideal, clinical replantation of limbs with 24 hours of cold ischemia could potentially lead to severe damage to the replanted tissue and host, which was ethically not acceptable. Second, although an effort was made to perform functional testing, our study lacks functional assessments, as our graft is heterotopic and nonfunctional. Third, this study encompasses a replantation model, which does not include a contralateral control and an analysis of the immunologic effects of prolonged preservation. Finally, this study does not provide a histologic analysis of the effects on skin, nerves, bones, or any other tissue, which has major relevance in VCAs. Nevertheless, this porcine replantation model has an increased translational relevance to clinical scenarios, due to the proportions of the limb, compared with small animal models.

The presented study is rooted in the fundamental principles of extended preservation and expands upon previous concepts using a custom MP setup and different acellular solution. Given the limited number of research groups focusing on this niche, particularly those investigating prolonged reperfusion outcomes, demonstrating reproducibility by independent researchers is crucial to validate preclinical observations in clinical trials. This study suggests the potential to extend the allowable ex vivo preservation duration by a factor of 4 to 6 times compared with existing standards. Such progress holds promise for substantially improving viability outcomes in both replantation and transplantation scenarios. By equipping ambulances with portable perfusion devices and readily accessible perfusate, tissues could be adequately preserved during transport to hospitals capable of transplantation or replantation after traumatic amputation. This strategy has the potential to ease the ongoing issue of donor shortages caused by logistical and time constraints during surgery, allowing for more comprehensive planning and preparation for the surgical procedure. Whereas SCS is affordable and simple to implement in clinics, MP is much more costly ($1100 per perfusion, calculated based on the costs of preservation fluid and disposables). These costs would need to be rationalized by improved outcomes and an increase in the chance for successful donor and recipient matching.

Future studies should establish a real-life approach protocol for ex situ MP of fully mismatched, heterotopic VCA in a porcine model, considering key parameters for graft viability. Standard heart–lung machines need adaptation, validation for long-term preservation effects on graft immunogenicity and survival, and global sharing with VCA physicians.

## CONCLUSIONS

MP for VCA is a promising technology that can overcome the limitations of cold storage, not only extending preservation times by sixfold but also enabling assessment of limb quality and allowing reconditioning of the limbs before transplantation. In this study, ex situ preserved porcine limbs in both groups demonstrated minimal degenerated muscle tissue 7 days after replantation.

## DISCLOSURE

The authors report no proprietary or commercial interest in any product mentioned or concept discussed in this article. There were no sources of funding for this work.

## ACKNOWLEDGMENTS

The authors thank Stephan van Raay, for providing artwork and digital supplements; Alex Hanssen, Maikel School, and Stefanie Schönfeld, for efforts throughout the project and animal caretaking; and Prof. Dr. Nens van Alfen and Sander Pagen, for setting up and helping with the nerve conduction studies.

## Supplementary Material


