# Supplementary figures and images for: 24-Hour Ex Vivo Hypothermic Acellular Perfusion of Porcine Forelimb: A 7-Day Follow-up Study
Source: Plast Reconstr Surg. 2024 Apr 15;154(6):1138–48. doi: 10.1097/PRS.0000000000011469 (PMC11584191; doi:10.1097/PRS.0000000000011469)

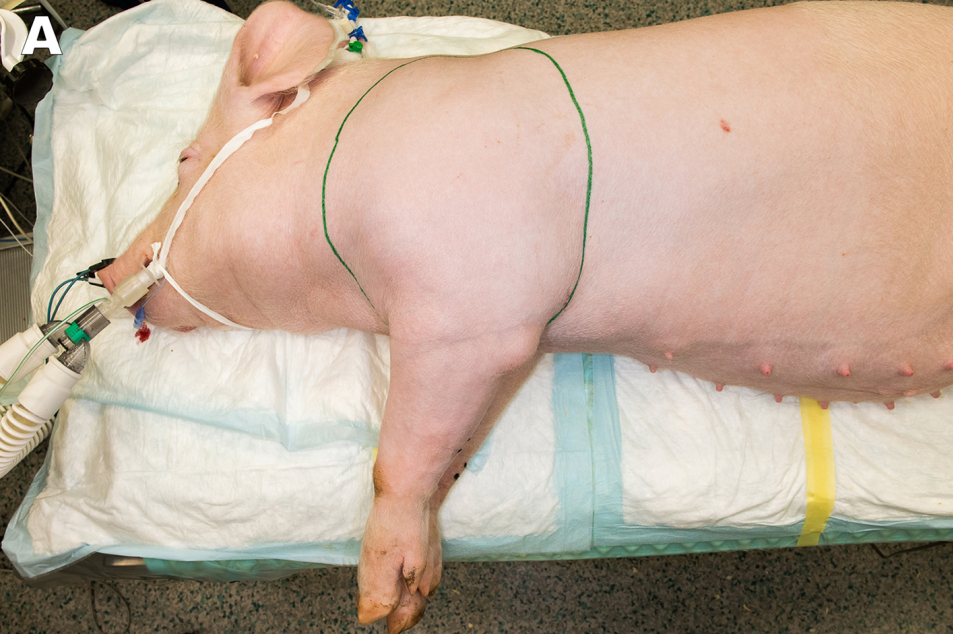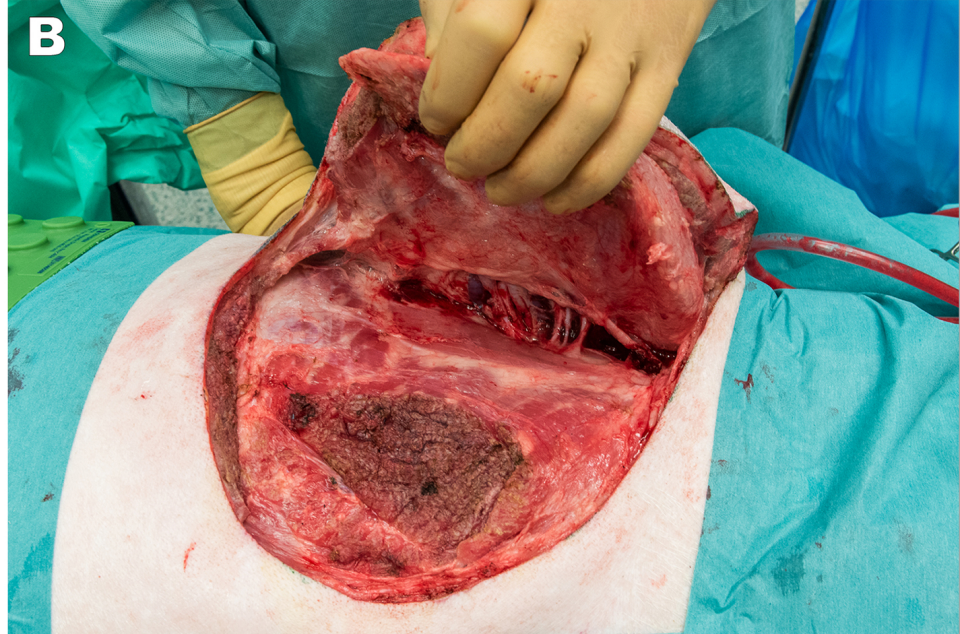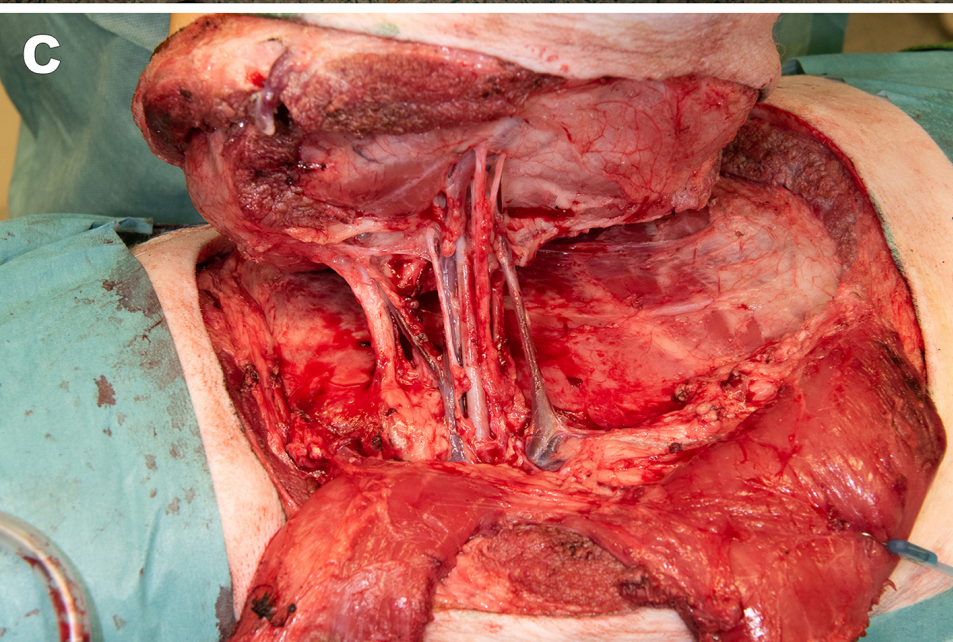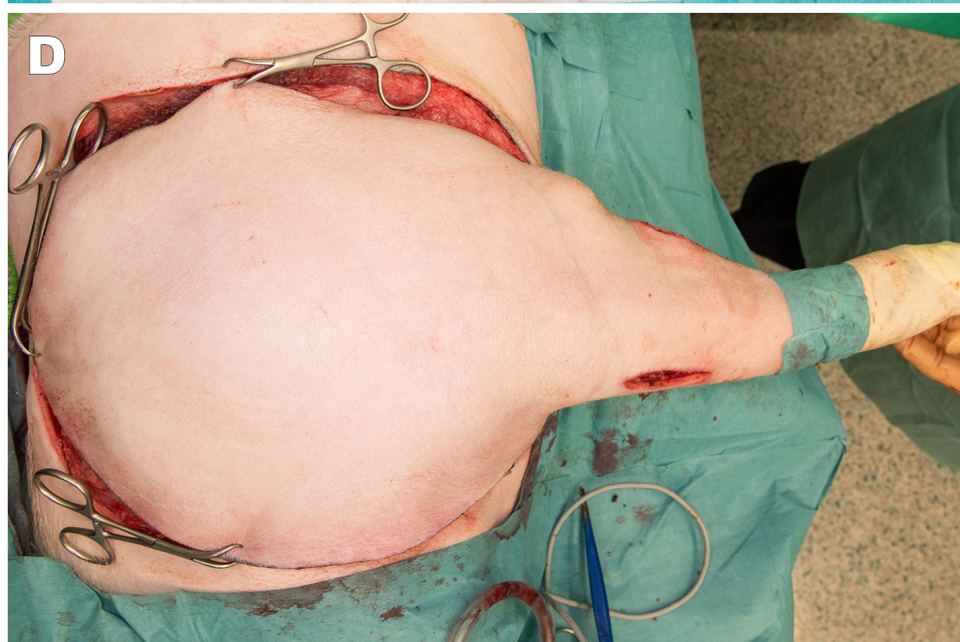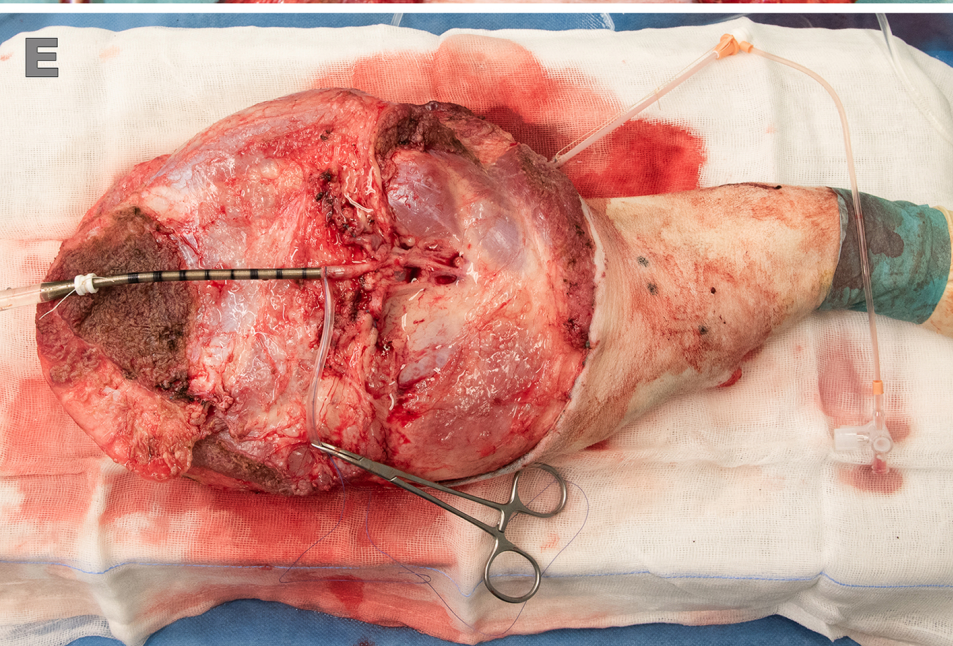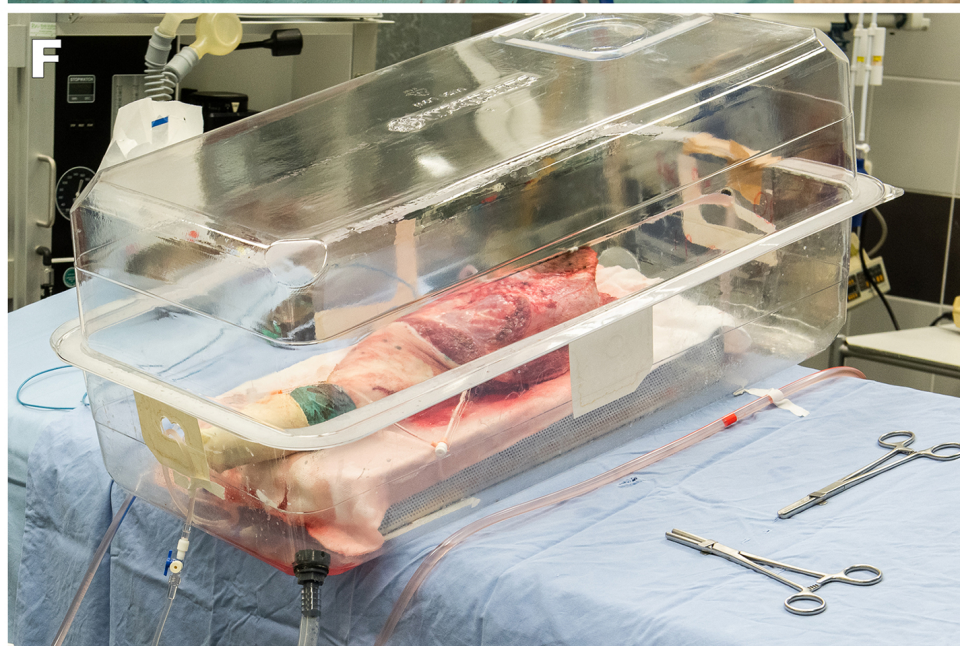

Supplement: Supplementary file 2 [file prs-154-1138e-s002.pdf]

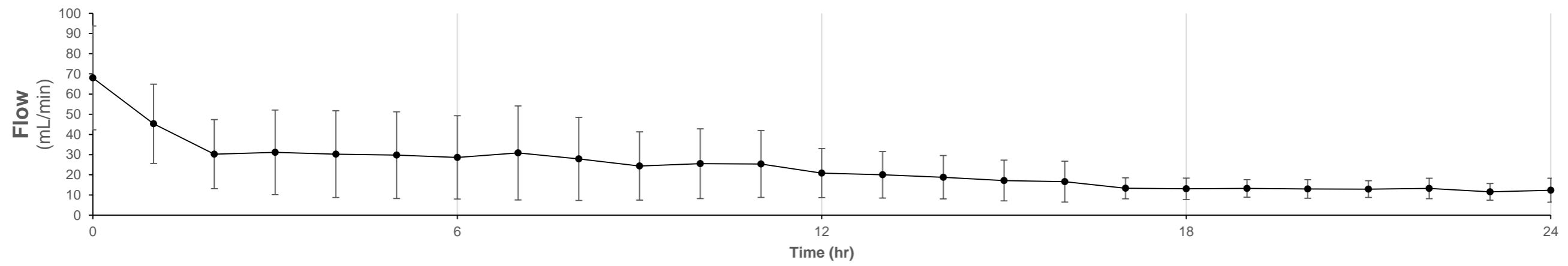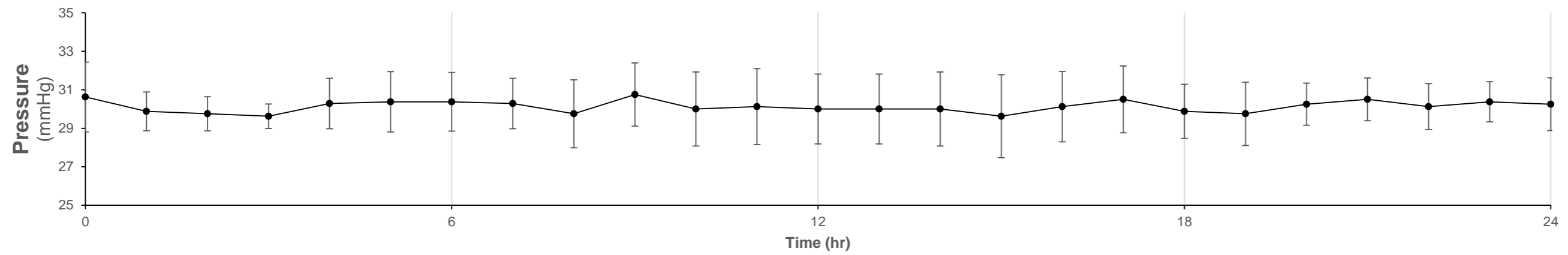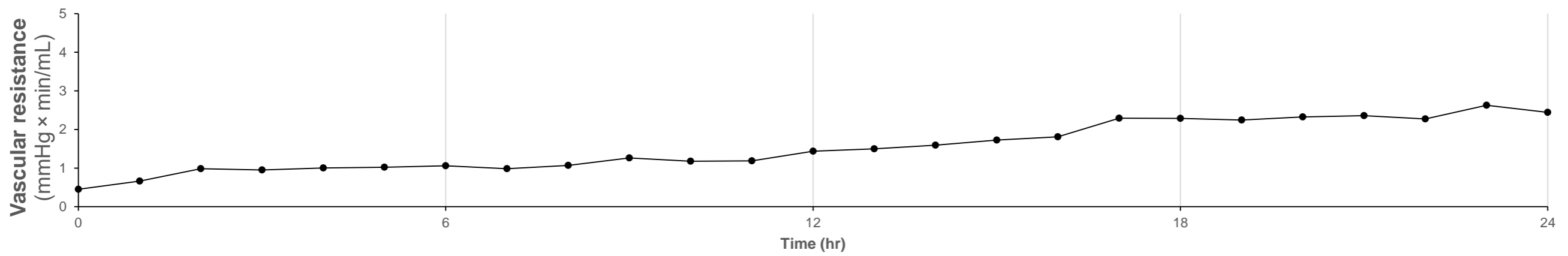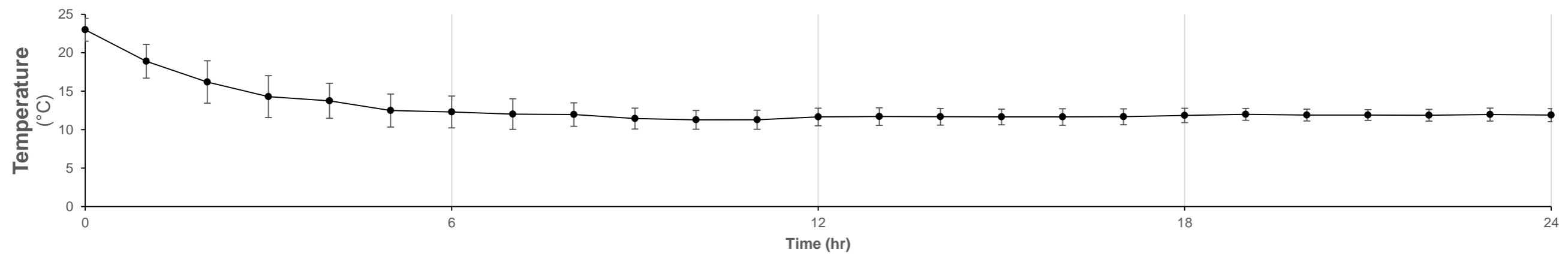

Supplement: Supplementary file 4 [file prs-154-1138e-s004.pdf]

Baseline

Replantation day 0

Replantation day 3

Replantation day 7

SCS

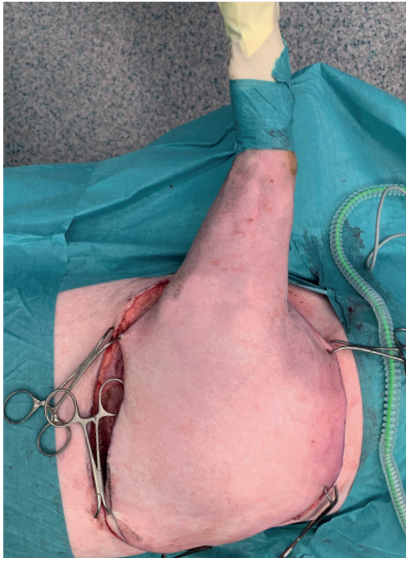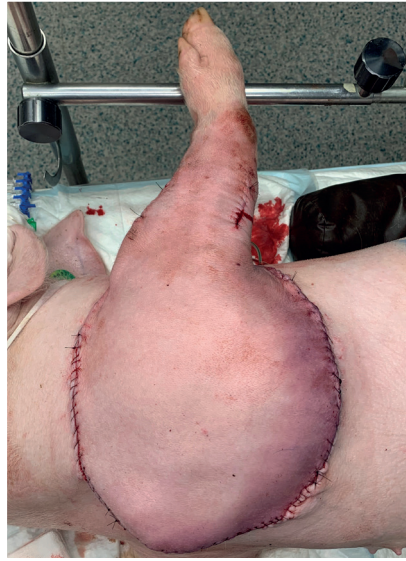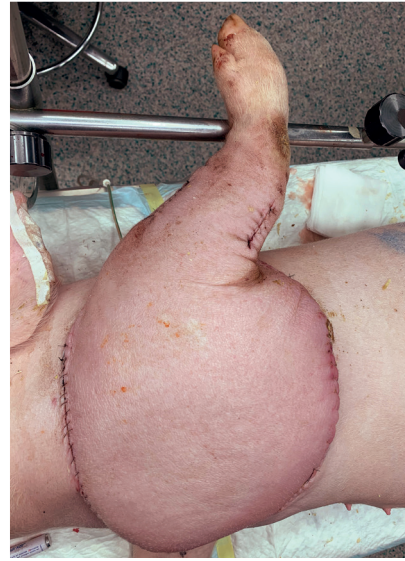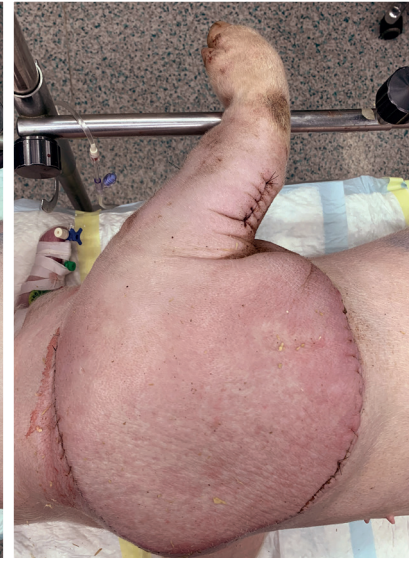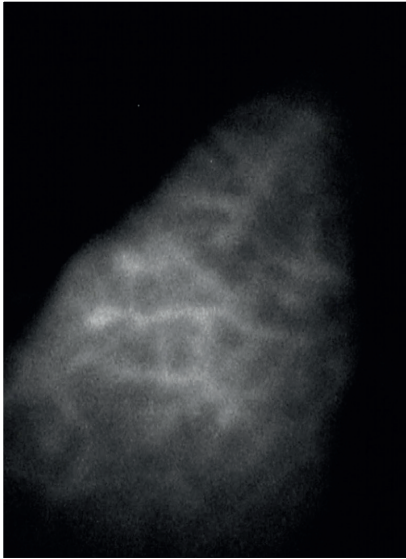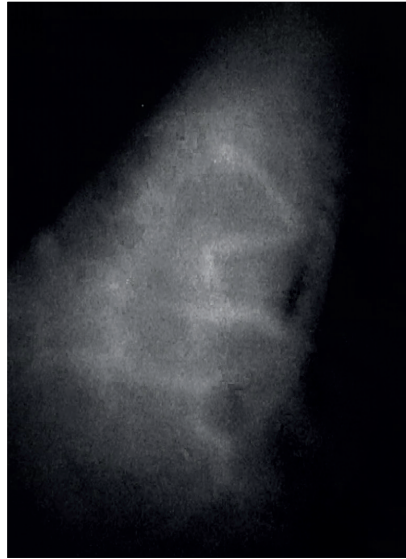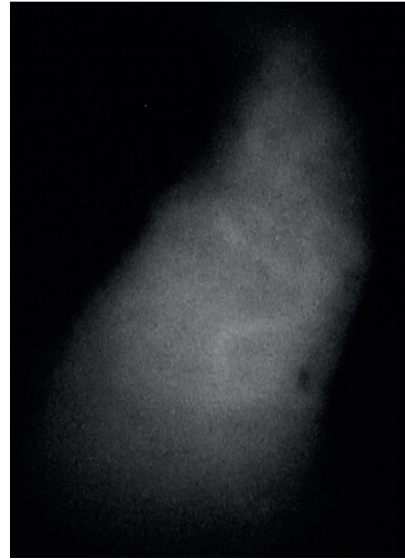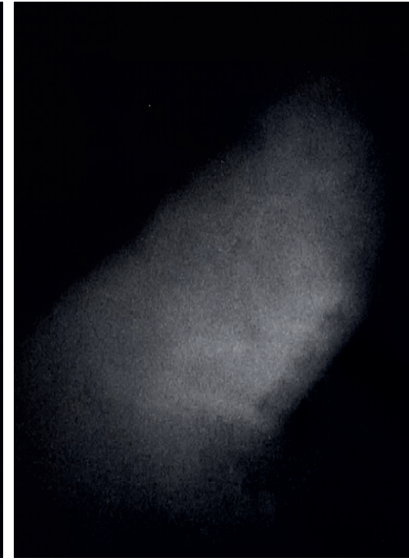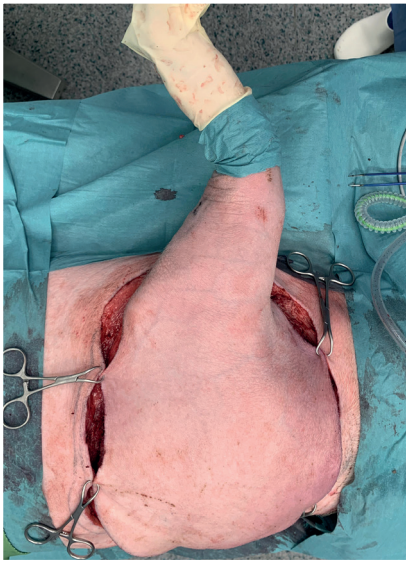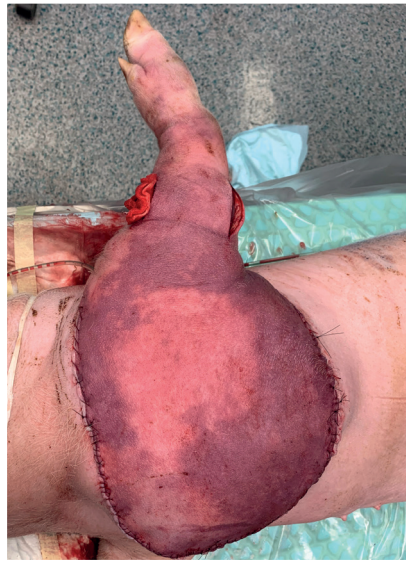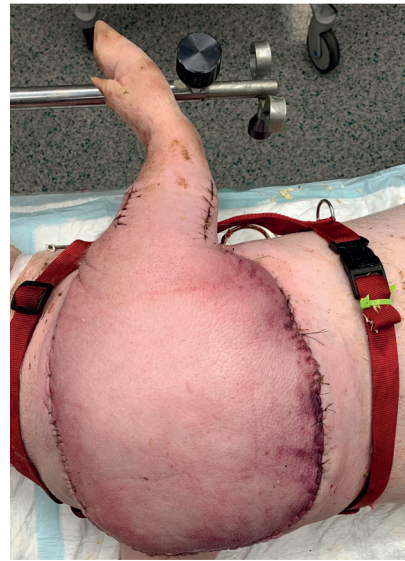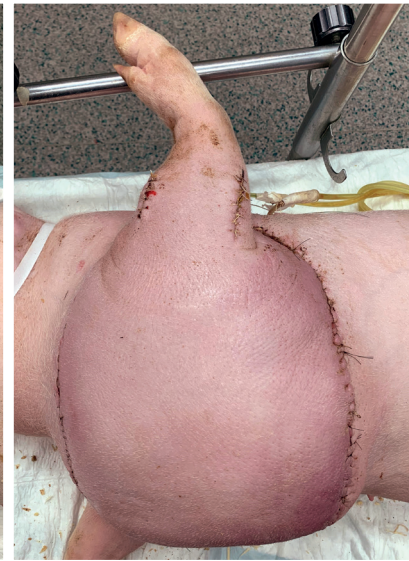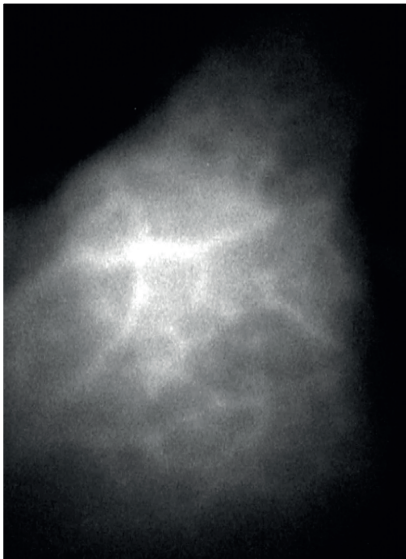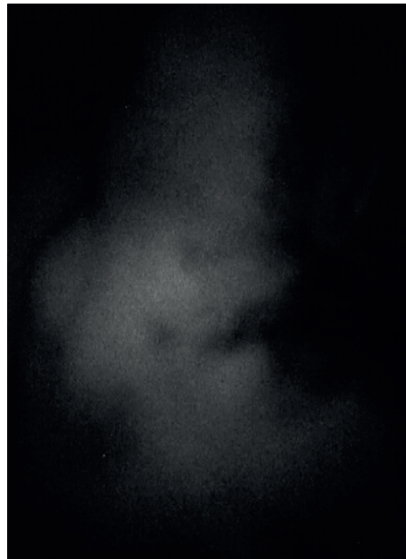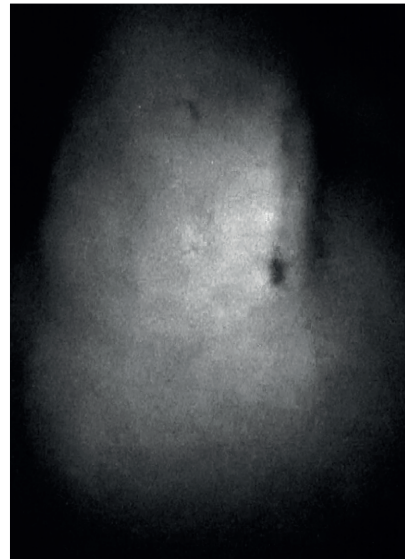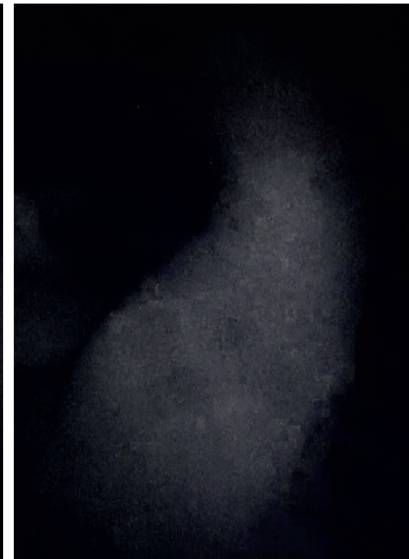

Perfusion

Supplement: Supplementary file 5 [file prs-154-1138e-s005.pdf]
