# Supplementary material for: 24-Hour Ex Vivo Hypothermic Acellular Perfusion of Porcine Forelimb: A 7-Day Follow-up Study
Source: Plast Reconstr Surg. 2024 Apr 15;154(6):1138–48. doi: 10.1097/PRS.0000000000011469 (PMC11584191; doi:10.1097/PRS.0000000000011469)
Supplement: Supplementary file 3 [file prs-154-1138e-s003.pdf]

	Viability assessments	Limb weight	NIR fluorescence angiography	Blood samples	Muscle biopsy	Perfusate aliquots (arterial + venous)	Perfusion parameters
Baseline	X	X	X	X	X		
During machine perfusion					Every 6 hours	Every 2 hours	Hourly
After ex-situ preservation / replantation	X	X	X	X	X		
Post-operative day 1	X			X	X		
Post-operative day 3	X		X	X	X		
Post-operative day 7	X	X	X	X	X		
